# The multistable melanopsins of mammals

**DOI:** 10.3389/fopht.2023.1174255

**Published:** 2023-04-26

**Authors:** Alan J. Emanuel, Michael Tri H. Do

**Affiliations:** F.M. Kirby Neurobiology Center and Department of Neurology, Boston Children’s Hospital and Harvard Medical School, Boston, MA, United States

**Keywords:** melanopsin (OPN4), opsin, pigment, bistable, tristable, bleaching, persistent activity, intrinsically photosensitive ganglion cell

## Abstract

Melanopsin is a light-activated G protein coupled receptor that is expressed widely across phylogeny. In mammals, melanopsin is found in intrinsically photosensitive retinal ganglion cells (ipRGCs), which are especially important for “non-image” visual functions that include the regulation of circadian rhythms, sleep, and mood. Photochemical and electrophysiological experiments have provided evidence that melanopsin has at least two stable conformations and is thus multistable, unlike the monostable photopigments of the classic rod and cone photoreceptors. Estimates of melanopsin’s properties vary, challenging efforts to understand how the molecule influences vision. This article seeks to reconcile disparate views of melanopsin and offer a practical guide to melanopsin’s complexities.

## Introduction

Organisms produce electrical responses to light in part by expressing photopigments, molecules that absorb photons, change conformation, and signal downstream. Over a thousand photopigments are known (1). Among them is melanopsin (2, 3). This molecule has features that are, presently, both unique and controversial. A unified understanding of these features is desirable because melanopsin exerts vital influences (4–7). For example, melanopsin helps synchronize the circadian clock to the solar day, thereby setting normal patterns of physiology and gene expression in practically all tissues of the body (8). Circadian dysregulation is implicated in disorders that range from mental illness to cancer (9–11). Melanopsin also plays roles in other species, such as fishes, frogs, lancelets, and reef corals (12). This review provides a practical synthesis of knowledge concerning the melanopsin molecules of mammals.

## The spectral sensitivity of melanopsin

**Figure 1:**
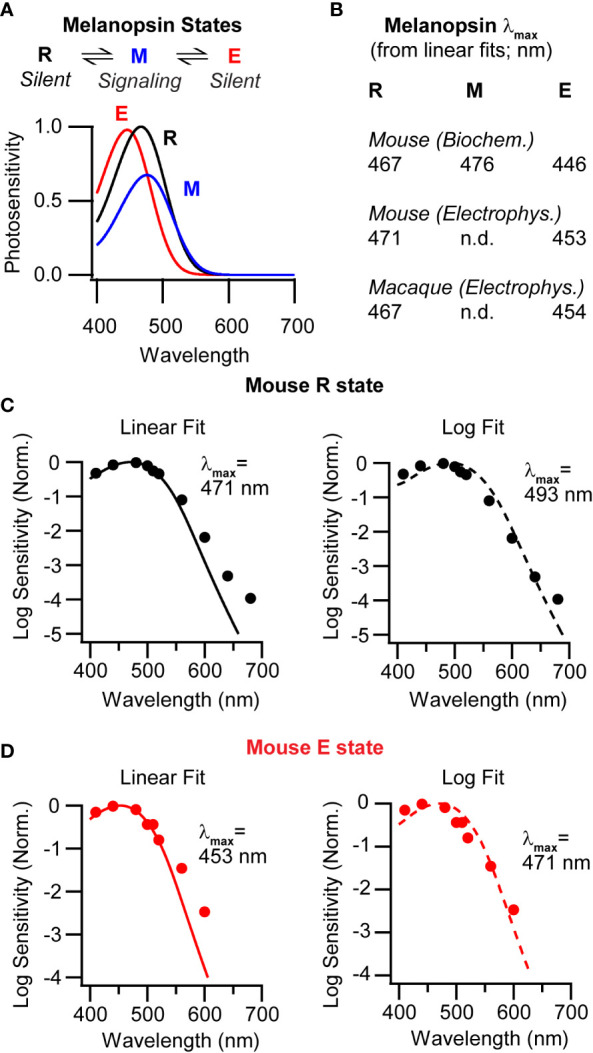
Melanopsin multistability in mice and macaques. **(A)** *Top*, Melanopsin states in the mouse. Melanopsin (R, the ground state, electrically silent) photoconverts with metamelanopsin (M, the signaling state), which photoconverts with extramelanopsin (E, electrically silent). *Bottom*, Photosensitivities of the three mouse melanopsin states (product of the extinction coefficient and quantum efficiency of isomerization) as a function of wavelength (20), normalized to that of the R state. **(B)** Wavelengths of peak sensitivity (λ_max_) for mouse melanopsin estimated by biochemistry and electrophysiology, and for macaque melanopsin estimated by electrophysiology (20–22). λ_max_ values were obtained by fitting nomograms on linear ordinates. **(C)** *Left*, Average of action spectra measured from mouse ipRGCs in darkness (markers) Data were previously published (21). The line is a single-state nomogram fit using a least-squares algorithm on a linear scale. Fit λ_max_ = 471 nm. *Right*, As on the left but the fit was made on a semi-log scale. Fit λ_max_ = 493 nm. **(D)** *Left*, Average of action spectra measured from ipRGCs during background illumination with 600-nm light to enrich for the E state (markers). The line is a single-state nomogram fit using a least-squares algorithm on a linear scale. Fit λ_max_ = 453 nm. *Right*, As on the left, but the fit was made on a semi-log scale. Fit λ_max_ = 471 nm.

A cardinal feature of a photopigment is its spectral sensitivity. Examining the literature, one has difficulty settling on the spectral sensitivity of mammalian melanopsin. Most estimates indicate that melanopsin is most sensitive to a wavelength near 480 nm (13–19). This wavelength of maximum sensitivity is referred to as the λ_max._ A single λ_max_ suggests that the molecule activates from a single state (or from multiple states that have the same spectral sensitivity). However, photochemical and electrophysiological measurements indicate that mouse melanopsin activates from two spectrally distinct states (**Figures 1A, B**) (20, 21). The ground state, melanopsin, abbreviated “R” for historical reasons, has a λ_max_ of ~470 nm. The second state, extramelanopsin (E), has a λ_max_ of ~450 nm. These values also apply to the melanopsin of macaque monkeys (22) (**Figure 1B**). Puzzlingly, none of these λ_max_ values are near 480 nm. Consideration of two factors offers a potential reconciliation.

First, the λ_max_ of a photopigment is often determined by fitting a discrete data set with a continuous function and taking the peak of that fit (23–25). These functions, often referred to as nomograms, are empirical and remarkably accurate (25). Given the λ_max_ of a pigment as the only free parameter, a nomogram describes the spectral sensitivity well. However, ambiguity arises in how the fit is performed. If sensitivity is plotted on a linear scale, the fit tends to weigh the peak sensitivity more heavily. If the response is plotted on a log scale, the fit tends to weigh the long-wavelength decline of sensitivity more heavily. The aforementioned photochemical and electrophysiological estimates of melanopsin’s spectral sensitivity (λ_max_ values of ~470 and ~450 nm) were made with fits on a linear scale. If one fits the electrophysiological data on a log scale, the λ_max_ values are red-shifted to ~490 nm for the R state and ~470 nm for the E state (**Figures 1C, D**). This ~20-nm disparity between linear and log fits is substantial. Which λ_max_ values to choose?

One might select according to context. A log fit might be undesirable because the long-wavelength decline of spectral sensitivity is labile. When wavelengths are longer than a certain value (λ_critical_, where λ_max_ = 0.84 λ_critical_), sensitivity has a more positive slope at higher temperature (26). On a log scale, fitting the R state’s action spectrum (21) using all data points or only those below λ_critical_ yields λ_max_ values of 493 and 476 nm, respectively. For the E state, these values are 471 and 463 nm. Repeating the exercise on a linear scale yields no difference for either state. On the other hand, a log fit might be desirable because the long-wavelength decline encompasses a broad range of tested sensitivities, unlike the peak. This trade-off likely explains why measurements vary across studies that have different emphases. Practical advice is to use the λ_max_ of the linear fit when short wavelengths are more relevant, and that of the log fit when long wavelengths are. Fits may also be made to portions of the data according to need (25).

**Figure 2:**
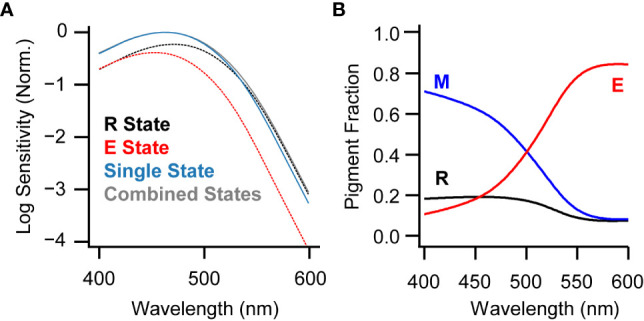
Melanopsin states under broadband and narrowband illumination. **(A)** Fits made to the action spectrum measured from ipRGCs on a background of broadband white light (21) using either a single-state nomogram (blue; λ_max_ = 463 nm when fit on linear scale) or the combination of the R and E spectra (gray; λ_max_ = 463 nm). Black and red dotted lines represent the R and E state nomograms, respectively, scaled to their contributions to the combined fit and using λ_max_ values measured electrophysiologically (471 and 453 nm, respectively). The λ_max_ of the combined nomogram, when fit on a log scale, is 480 nm. **(B)** Melanopsin states at photoequilibrium for monochromatic illumination with wavelengths spanning the visible range, predicted by a model based on biochemical parameters of mouse melanopsin (21).

The second consideration is that melanopsin equilibrates among its three known states during illumination (20, 21). Under common lighting conditions, the population of melanopsin molecules activates about equally from the R and E states (21). Taking the λ_max_ values of these states as 450 and 470 nm (from fits on a linear ordinate), one obtains an effective λ_max_ of ~460 nm (**Figure 2A**). Using log ordinate fits instead, where λ_max_ values are 470 and 490 nm, the effective λ_max_ is ~480 nm. Most studies of melanopsin’s spectral sensitivity use stimuli that are sufficiently long and intense that they are likely to produce an equilibrium of states. Thus, the commonly observed λ_max_ of 480 nm can be explained by melanopsin’s multistable nature and the popularity of fitting on a log ordinate.

Melanopsin’s activation from two spectrally distinct states causes the population of molecules to have a broader spectral sensitivity than a single state (21). This broadening may be considered ample, as the difference in λ_max_ values between the R and E states is roughly comparable to that between the long- and medium-wavelength sensitive cone pigments that give rise to the red-green color axis in humans (27). Indeed, a broadened spectral sensitivity is consistent with the role of melanopsin in sensing the overall light intensity and not necessarily specific wavelengths (but see 28). On the other hand, this broadening may be considered slight. Consider the predicted activation of melanopsin in sunlight (CIE spectrum G174), comparing the realistic case of a combined-state spectral sensitivity and the hypothetical case of a single-state spectral sensitivity, each having a λ_max_ value of 480 nm. The combined-state, broader spectrum absorbs ~3% more than the single-state, narrower spectrum. It would appear that, at least under sunlight, the spectral broadening caused by melanopsin multistability is subtle. Nevertheless, in cases that require precision, it is not much trouble to use the combined-state spectrum rather than its single-state approximation.

To conclude this section, it may be sufficient in most contexts to approximate melanopsin’s spectral sensitivity with a standard Govardovskii nomogram that has a λ_max_ of 480 nm (on a log scale) or 460 nm (on a linear scale). Accommodating additional complexity increases accuracy.

## Specific features of melanopsin multistability

At this point, it appears that melanopsin can be considered in relatively simple terms. However, there are cases where specific features of melanopsin multistability are especially salient. This section will highlight five.

### Persistent activity

Melanopsin’s signaling state, metamelanopsin (M), is subject to termination mechanisms that include phosphorylation, arrestin binding, bleaching, and internalization (29–39). Nevertheless, some melanopsin signaling endures and drives persistent cellular activation (21, 22, 40, 41). This activity is consistent with the melanopsin system’s encoding of environmental irradiance. Prolonged activation tends to blur spatial and temporal details in the scene, emphasizing the overall light intensity (21, 22). The overall light intensity is information that is used, for instance, by the circadian clock for synchronization to the day/night cycle (42). Persistent melanopsin activity is also thought to drive the post-illumination pupil response (PIPR), which has been used to diagnose the melanopsin system in contexts ranging from seasonal affective disorder to Alzheimer’s disease (41).

### Photoswitching

As mentioned above, photon absorptions interconvert melanopsin among its states. Sunlight and most common sources of artificial white light produce a photoequilibrium in which roughly half the melanopsin molecules are in M state and a quarter each are in the R and E states. Photoswitching maintains a pool of melanopsin molecules for activation, as those that are driven from the M state are available for reactivation (21, 22, 43).

The photoequilibrium fractions of melanopsin can be manipulated using narrow-band light (**Figure 2B**) (20, 21). The M state, having the longest λ_max_, dominates under short wavelengths and is minimal under long wavelengths. The E state, having the shortest λ_max_, follows the opposite pattern (while the R state has an intermediate λ_max_ and only a small fraction is present after exposure to any wavelength). This trade between M and E states is reflected in the magnitude of persistent activity in ipRGCs. Short wavelengths produce the largest persistent activity and long wavelengths the smallest (21, 22). Thus, ipRGC activation can be switched high and low with acute illumination with short and long wavelengths. Photoswitching of persistent activity has been demonstrated for mouse and macaque ipRGCs (21, 22), as well as for cell lines expressing human melanopsin (40).

Practically speaking, deactivation of ipRGCs requires intense and prolonged illumination because all melanopsin states absorb long wavelengths poorly. The optimal wavelength for deactivation is near 560 nm and reflects a balance between being long enough for preferential absorption by the M state but not so long that it is scarcely absorbed at all (21). At 560 nm, deactivation can be produced by delivering ~10^9^ photons µm^-2^ sec^-1^ for 30 s or more (21, 22). This kind of light is probably not found in nature, though artificial sources are available.

### Bleaching and regeneration

Bleaching is the process by which an activated photopigment dissociates into opsin and chromophore (44). Neither opsin nor chromophore absorb well in the visible spectrum so this process causes the appearance of an actual bleach to the human eye. For example, a solution of rhodopsin—a molecule once called visual purple—loses its color in light. Bleaching and multistability are not mutually exclusive. Thus, though active melanopsin is stable and can be photoconverted to a silent state, it can also release its chromophore (36, 37, 39, 45). Indeed, applying exogenous chromophore to ipRGCs increases their sensitivity, though it is unclear if this effect is due to natural levels of bare opsin or bleaching during the course of the experiment (36, 45, 46).

The only chromophore found in dark-adapted ipRGCs is 11-*cis* retinal (47), which defines melanopsin’s R state (20). Curiously, there appears to be no kind of illumination that produces only the R state in ipRGCs. As mentioned above, this state seems sparse during illumination with any spectrum (21). Therefore, a light-independent pathway should recover melanopsin to the R state. Evidence exists for chromophore supply from the retinal pigment epithelium to ipRGCs *via* Müller glia cells (48) even though questions remain (49). Perhaps the process of dark regeneration involves melanopsin bleaching and 11-*cis* retinal resupply.

### Adaptation

Melanopsin activity drives adaptive processes that tamp down on melanopsin activity (29–39, 50–52). Consequently, melanopsin deactivation may reverse adaptation to produce sensitization. Sensitization of this kind has been suggested by *in vivo* experiments (53). Anecdotal evidence can be found in ex vivo experiments as well (21). Melanopsin phototransduction also drives light adaptation at the level of the ipRGC population (54). The interplay of activation and adaptation in the melanopsin system merits further study.

### Species variation

At least two stable, silent states of melanopsin have been observed in mice and macaques (21, 22). In humans and the lancelet, amphioxus, only one silent state has been reported (55, 56). Thus, the evolutionary conservation of melanopsin multistability may be incomplete. Also, across species, melanopsins vary in their bleaching rates (37). This variation may influence the lifetimes of persistent responses across species. Indeed, mouse melanopsin (reluctant to bleach) and human melanopsin (willing to bleach) have relatively long and brief persistent responses, respectively (37, 40). Another layer of intricacy is that persistent response lifetime may be modulated, melanopsin can be alternatively spliced, melanopsins are functionally diverse, and melanopsins show sufficient molecular distinctions to be grouped into two gene families (Opn4m and Opn4x) (57–63). Further study may reveal additional diversity in melanopsins across species.

## Closing remarks

Melanopsin’s discovery indicated that the mammalian retina is not duplex—relying on rods and cones to sense light—but multiplex. The multiplicity of melanopsin states adds richness to this picture. This review intends to provide a concise summary of melanopsin’s complexities and a practical guide on how to navigate them.

## Author contributions

Writing and figures: MTHD. Data, analyses, figures, and critiques: AJE. All authors contributed to the article and approved the submitted version.
